# Individual and contextual-level factors associated with iron-folic acid supplement intake during pregnancy in Ethiopia: a multi-level analysis

**DOI:** 10.1186/s12884-023-05593-7

**Published:** 2023-04-18

**Authors:** Melaku Yalew, Shiferaw Getachew, Keriya Mohammed, Hailu Hankarso, Adane Bayile, Shambel Dessale Asmamaw, Mesfin Getahun Assefa, Getaw Walle Bazie, Wondwosen Mebratu, Bereket Kefale, Yitayish Damtie, Mastewal Arefaynie, Tesfaye Birhane, Reta Dewau, Nigus Cherie, Elsabeth Addisu, Kefale Mitiku, Fentaw Tadese, Teklehaimanot Fentie Wendie, Adane Habtie, Tefera Chane Mekonnen, Sisay Eshete Tadesse, Getachew Tadesse Bedane, Yitbarek Wasihun, Tilahun Degu Tsega, Mekuanint Taddele, Zenebe Tefera, Bezawit Adane, Birhanu Wagaye, Fanos Yeshanew Ayele, Aregash Abebayehu Zerga, Abebaw Molla, Biruk Desalegn, Mengesha Birkie, Bekalu Bewket, Belete Kassa Alemu, Segenet Zewdie, Meseret Kefale Tsegaye, Abebayehu Bitew, Kassu Mehari, Lemma Derseh

**Affiliations:** 1 Department of Epidemiology and Biostatistics, School of Public Health, College of Health Sciences, Injibara University, Injibara, Ethiopia; 2 Department of Reproductive and Family Health, School of Public Health, College of Medicine and Health Sciences, Wollo University, Dessie, Ethiopia; 3 Head office manager of Mida Weremo woreda, North Shewa, Amhara Regional State Ethiopia; 4 Independet researcher, Dessie, Amhara Regional State Ethiopia; 5 Yirgalem Medical College, Hawassa University, Hawassa, Ethiopia; 6 Head manager of Maternal and Child Health, Boru Meda Hospital, Amhara Regional State, Bahir Dar, Ethiopia; 7 Head manager of Kobo Primary Hospital, Amhara Regional State, Bahir Dar, Ethiopia; 8 Safty, Health and Environmental Manager at CEVA Logistics, Addis Ababa, Ethiopia; 9 Department of Epidemiology and Biostatistics, School of Public Health, College of Medicine and Health Sciences, Wollo University, Dessie, Ethiopia; 10 Department of Reproductive and Family Health, School of Public Health, College of Health Sciences, Injibara University, Injibara, Ethiopia; 11 Department of Physiology, College of Health Sciences, Injibara University, Injibara, Ethiopia; 12 Department of Pharmacy, College of Medicine and Health Sciences, Wollo University, Dessie, Ethiopia; 13 Department of Health Promotion, College of Medicine and Health Sciences, Wolkite University, Wolkite, Ethiopia; 14 Department of Nutrition and Dietetics, School of Public Health, College of Medicine and Health Sciences, Wollo University, Dessie, Ethiopia; 15 Department of Statistics, College of Natural Sciences, Wollo University, Dessie, Ethiopia; 16 Department of Health Promotion, School of Public Health, College of Medicine and Health Sciences, Wollo University, Dessie, Ethiopia; 17 Department of Health Promotion and Behavior, School of Public Health, College of Health Sciences, Injibara University, Injibara, Ethiopia; 18 Department of Midwifery, School of Nursing and Midwifery, College of Medicine and Health Sciences, Wollo University, Dessie, Ethiopia; 19 Department of Public Health Nutrition, College of Medicine and Health Sciences, Injibara University, Injibara, Ethiopia; 20 Department of Epidemiology and Biostatistics, Kotebe Health Science College, Kotebe University, Addis Abab, Ethiopia; 21 Department of Psychiatry Nursing, College of Medicine and Health Sciences, Injibara University, Injibara, Ethiopia; 22 Department of Adult Health Nursing, College of Medicine and Health Sciences, Injibara University, Injibara, Ethiopia; 23 Department of Pharmacy, College of Medicine and Health Sciences, Injibara University, Injibara, Ethiopia; 24 Department of Epidemiology and Biostatistics, School of Public Health, College of Medicine and Health Sciences, Bahir Dar University, Bahir Dar, Ethiopia; 25 Department of Gynecology and Obstetrics, School of Medicine, College of Medicine and Health Sciences, Bahir Dar University, Bahir Dar, Ethiopia; 26 Department of Epidemiology and Biostatistics, Institute of Public Health, College of Medicine and Health Sciences, University of Gondar, Gondar, Ethiopia

**Keywords:** Iron intake, Iron supplement, Anemia, Pregnant women, Ethiopia

## Abstract

**Background:**

Anemia is still one of the major public health problems in many developing countries including Ethiopia. Thus, this study aimed to assess individual and contextual-level factors associated with iron-folic acid supplement intake during pregnancy in Ethiopia.

**Methods:**

A secondary analysis was done on the 2019 mini-Ethiopian Demographic and Health Survey (EDHS) dataset. A total of 3,927 pregnant women who gave birth five years before the survey were included in the analysis. Multi-level mixed-effect logistic regression analysis was done by STATA/SE version 14.0 to identify individual and contextual-level factors. Adjusted Odds Ratio (AOR) with 95% Confidence Interval (CI) was used to show the strength and direction of the association. The level of statistical significance was declared at a P value less than 0.05.

**Results:**

Those primary educated [AOR **=** 1.83, 95% CI: (1.24, 2.74)], secondary educated [AOR **=** 2.75, 95% CI: (1.57, 4.824)], women who had greater than 5 living children [AOR = 2.02, 95% CI: (1.25, 3.27)], women who had ANC visit [AOR = 21.26, 95% CI: (13.56, 33.32)] and women who lived in a cluster with high proportion of women had ANC visit [AOR = 1.72, 95% CI: (1.17, 2.54)] and women who lived in Somali [AOR = 0.44 0.73, 95% CI: (0.22, 0.87)] were significantly associated with iron-folic acid intake during pregnancy.

**Conclusions:**

Both individual and contextual-level factors were significantly associated with iron-folic acid intake during pregnancy. From individual-level factors: education status of women, the total numbers of living children, and ANC follow-up are significant and from contextual-level factors: region and living in a high proportion of women who had ANC follow-up were found to have a statistically significant association. Promoting women’s education and maternal health services like ANC and intervention targeting the Somali region would be the recalled area of the government.

## Background

The global estimate indicated that nearly 42% of pregnant women and 30% of non-pregnant women are found to be anemic [1, 2]. In Sub-Sahara Africa, 20% of maternal mortality was directly attributed to anemia [1]. According to 2016, Ethiopia Demographic and Health Survey, 24% of reproductive-age women were anemic [3]. Anemia among reproductive-age women who reside in rural and urban areas was 25% and 17%, respectively [4]. In several low-income countries, the cause of anemia during pregnancy is multi-factorial. It includes nutrition deficiencies of folate, vitamin B12, iron, and parasitic diseases such as malaria and hookworm [5].

Anemia develops as a result of ineffective or deficient erythropoiesis or loss of red blood cells and iron deficiency anemia (IDA) and anemia of red blood cell morphology is types of anemia that have different biological mechanisms of causation. Iron in the human body is regulated by the iron cycle and most of the storage iron human body is used for new red blood cell (RBC) synthesis or erythropoiesis and the lack of iron stored in the body limits the erythropoiesis process which leads to a reduction in the number of circulating RBCs lower than normal and insufficient oxygen-carrying capacity to meet physiological demands subsequently results in the development of IDA [6, 7]. On the other hand, folate has a crucial role in the DNA synthesis pathway and the deficiency of folate or vitamin B12 affects DNA synthesis and cell division in the bone marrow (megaloblastic changes). This deficiency leads to nuclear division without significant alteration in the cytoplasmic maturation cycle and nucleated precursor cells in the bone marrow develop immature or morphologically abnormal nuclei and giant metamyelocytes, with macrocytic red blood cells called megaloblastic anemia [8].

Iron and folic acid deficiencies are the most common causes of anemia among pregnant women in sub-Saharan Africa. Iron deficiency anemia contributes to adverse effects on maternal and child health. The maternal consequences include: low weight gain, preterm labor, placenta previa, premature rupture of membrane, cardiac arrest, hemorrhage, lowered resistance to infection, poor cognitive development, and reduced work capacity. But, it is not limited to the above-mentioned effects. Similarly, it has fetal and neonatal risks including prematurity, low birth weight, and fetal distress, which contributes to perinatal morbidity and mortality. Infants born to anemic women are more likely to become anemic [9, 10]. Folic acid (the synthetic form of Vitamin B9 or folate) deficiency at conception and in early pregnancy is also associated with an increased risk of neural tube defect, preeclampsia, fetal malformations, and preterm delivery [11, 12].

In Ethiopia, IFA supplementation is one of the main strategies for the prevention of anemia. The Ethiopian national guideline for the prevention of micronutrient deficiencies recommended the need for daily IFA supplementation during pregnancy and postpartum. However, the effectiveness and success of such interventions depend on the adherence to IFA tablets [13].

Even though iron-folic acid intake or supplementation among pregnant women was addressed in previous studies, most of them were taking on individual-level analysis by omitting the effect of clustering. In the classical logistic regression (individual-level analysis), the independent assumption among clustered individuals is violated. In addition, the association at the individual-level may not work at the cluster-level and vice versa. So, most of the previously published articles are subject to an atomistic or ecological fallacy [14–19]. The factors associated with iron-folic acid supplement intake are area-specific which requires a different approach to analysis at a different level [20, 21]. Therefore, this study took into account those different levels of analysis and aimed to assess individual and contextual-level factors associated with iron-folic acid supplement intake among pregnant women in Ethiopia by using the mini-EDHS 2019 dataset.

## Methods

### Study area and data source

**Fig. 1:**
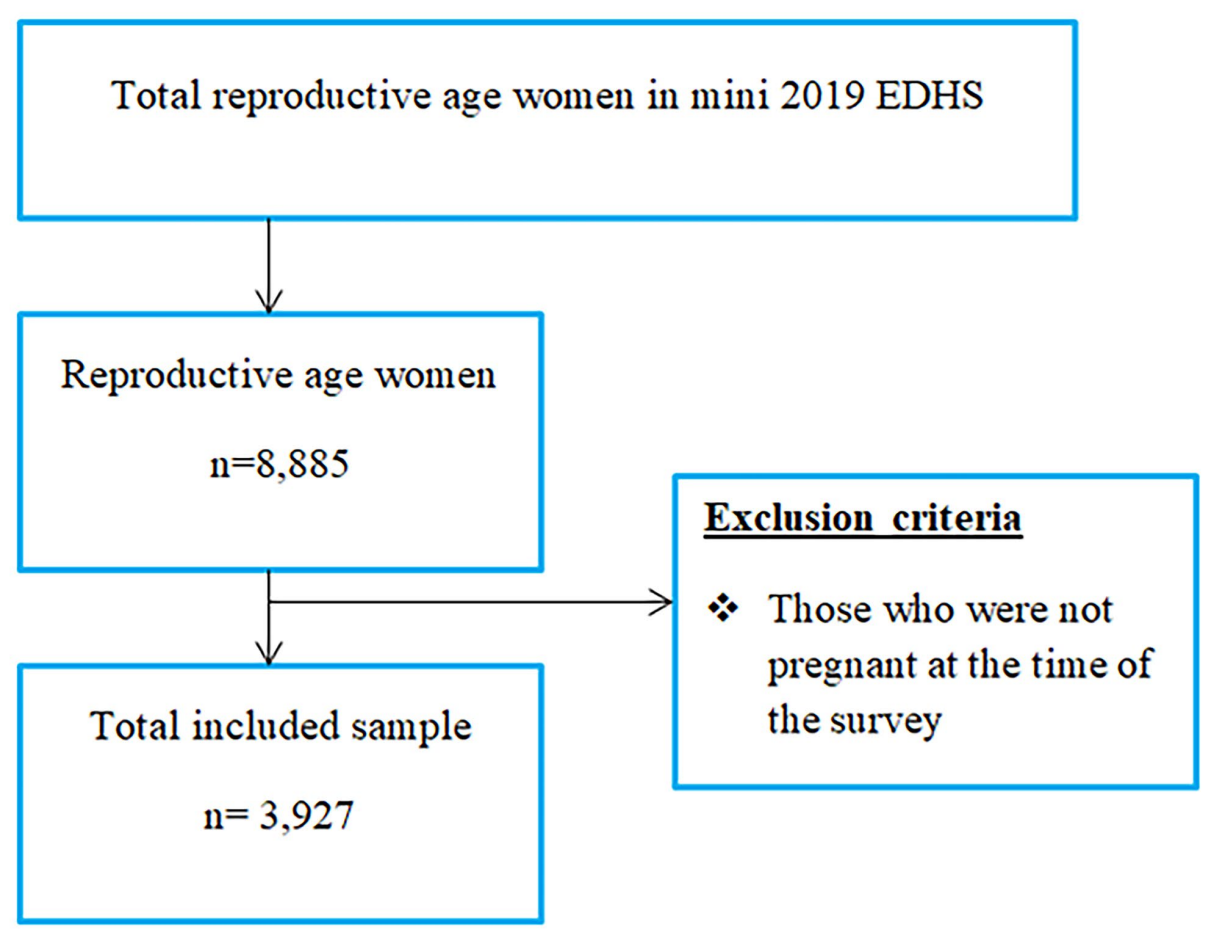
Sample size and sampling procedure to reach the study participants in mini-EDHS 2019, Ethiopia

The study was conducted in Ethiopia, which is located in the North-Eastern part of Africa, which lies between 3^0^ and 15^0^ North latitude and 33^0^ and 48^0^ East longitudes. This study used the 2019 mini-EDHS dataset. It was collected by the Central Statistical Agency (CSA) in collaboration with the Federal Ministry of Health (FMoH) and the Ethiopian Public Health Institute (EPHI). Data were accessed from their URL: www.dhsprogram.com by contacting them through personal accounts after justifying the reason for requesting it [22]. A cross-sectional study design using secondary data from the 2019 mini-EDHS was conducted. A total of 3,927 weighted pregnant women were included in the analysis (Fig. 1).

### Variable measurement, data processing, and analysis

The outcome variable for this study is dichotomized as iron-folic acid supplement intake (yes/no) which was generated from a constructed mini-EDHS variable. Wealth index is a composite variable that was calculated for urban and rural separately and it includes the following classification [3].

- **Poorest**: Includes women whose wealth was less than or equal to 20 percentile.
- **Poorer**: Includes women whose wealth ranged 21 to 40 percentile.
- **Middle**: Includes women whose wealth ranged 41 to 60 percentile.
- **Richer**: Includes women whose wealth ranged 61 to 80 percentile.
- **Richest**: Includes women whose wealth ranged 81 to 100 percentile.

Contextual-level variables were created by taking aggregate measures from individual-level variables in each cluster. Data cleaning was conducted to check for consistency and missing value. Recoding, labeling, and exploratory analysis were performed by using Stata/SE version 14.0. Sample weight was used to compensate for the unequal probability of selection between the strata that were geographically defined, as well as for non-responses [23]. Descriptive statistics were used to present frequencies, with percentages in tables, graphs, and using texts. Multilevel analysis was conducted after checking that the data was eligible for multilevel analysis which means Intra-cluster Correlation Coefficient (ICC) was high (ICC = 32.04%). Since DHS data are hierarchical, i.e. individuals (level 1) were nested within communities (level 2 or cluster), a two-level mixed-effects logistic regression model was fitted to estimate both independent (fixed) effects of the explanatory variables and community-level random effects on iron-folic acid intake [24]. The log of the probability of iron-folic acid intake was modeled using a two-level multilevel model as follows:

$$Log\ [\frac{{\Pi ij}}{{1 - \Pi ij}}] = {\beta _0} + {\beta _1}{X_{ij}} + {B_2}{Z_{ij}} + {\mu _j} + {e_{ij}}$$

Where, i and j are the level 1 (individual) and level 2 (contextual) units, respectively; X and Z refer to individual and contextual-level variables, respectively; πij is the probability of iron-folic acid intake for the i^th^ women in the j^th^ cluster; the β’s indicates the fixed coefficients. Whereas, β0 is the intercept-the probability of iron-folic acid intake in the absence of influence of predictors; and uj showed the random effect (effect of contextual factors on iron-folic acid intake for the j^th^ cluster and eij showed random errors at the individual levels. By assuming each cluster had a different intercept (β0) and fixed coefficient (β), the clustered data nature and the within and between cluster variations were taken into account. During analysis first, bivariable multilevel logistic regression was fitted and variables with a p-value less than 0.25 were selected to build the 3 models (model1-3). Then the analysis was performed in three steps: Model 1 (empty model or null model/ without explanatory variable); Model 2 (only individual-level factors) and Model 3 (both individual and contextual-level factors). The measures of association (fixed-effects) estimate the associations between the likelihood of women to take iron-folic acid and various explanatory variables were expressed as Adjusted Odds Ratio (AOR) with their 95% confidence level. A variable in which it’s a p-value < 0.05 was used to declare statistical significance. Intra-cluster Correlation coefficient (ICC), Median Odds Ratio (MOR), and proportional change in variance (PCV) were used to measure the variation between clusters. MOR is defined as the median value of the odds ratio between the area at highest risk and the area at the lowest risk when randomly picking out two areas. PCV measures the total variation attributed to individual-level factors and area-level factors in the multilevel model. The log-likelihood ratio test was used to estimate the goodness of fit of the adjusted final model in comparison to the preceding models.

## Results

### Socio-demographic characteristics of the respondents

The total numbers of pregnant women who gave birth within five years before the survey and included for analysis were 3,927. Out of this, 1,192 (30.37%) were founded in the age group 25–29 years. About 3,685 (93.84%) were currently married or living together. Of the total pregnant women, 2,014 (51.30%) had no education. Regarding household wealth, out of the total 825 (21.02%) lived in the poorest wealth quintile. Among responded mothers, 1,440 (36.69%) are orthodox in religion.

Regarding ANC visits, 1,013 (25.81%) of women had no ANC visits. Out of the total study participants, 2,900 (73.86%) resided in a rural area. About, 1,519 (38.69%) resided in Oromia region, and 839 (21.38%) resided in Amhara region. Concerning the total number of living children, about 1,539 (39.17%) women had 3–5 children (Table 1).

**Table 1:** Characteristics of pregnant women in Ethiopia who gave birth within 5 years before the survey, mini-EDHS 2019

Variables	Category	Frequency	Percentage
**Age in years**	15–19	227	5.79
	20–24	768	19.58
	25–29	1912	30.37
	30–34	799	20.36
	35–39	590	15.04
	40–44	258	6.59
	45–49	89	2.27
**Marital status**	Single	241	6.16
	Married	3684	93.84
**Place of residence**	Urban	1026	26.14
	Rural	2900	73.86
**Educational status of a mother**	No education	2014	51.30
	Primary	1414	36.03
	Secondary	344	8.79
	Higher	152	3.89
**Wealth**	Poorest	2,843	63.34
	Poorer	1,646	36.66
	Middle	1,924	45.35
	Richer	1,710	40.29
	Richest	393	9.27
**Religion**	Orthodox	1440	36.69
	Muslim	1339	34.12
	Protestant	1082	27.57
	Others	63	1.62
**ANC visit**	No	1013	25.81
	Yes	2913	74.19
**Total numbers of living child**	< 3	1583	40.33
	3–5	1537	39.17
	> 5	805	20.51
**Region**	Tigray	286	7.3
	Afar	51	1.3
	Amhara	839	21.38
	Oromia	1519	38.69
	Somali	210	5.55
	Benishangul	47	1.2
	SNN	787	20
	Gambela	19	0.49
	Harari	11	0.28
	Addis Ababa	126	3.22
	Dire Dewa	21	0.54
**Cluster ANC follow up**	Low	1818	46.31
	High	2108	53.69
**Cluster below middle wealth**	Low	1970	50.19
	High	1955	49.81
**Cluster secondary above education**	Low	2515	64.05
	High	1411	35.95

**Footnote**: Cluster ANC follow up = Proportion of women who had ANC in a cluster, Cluster below middle wealth = Proportion of women blow the middle wealth quartile in a cluster, and Cluster above secondary = Proportion of women educated secondary and above in a cluster

### Individual and contextual-level factors associated with iron-folic acid supplement intake (fixed-effects)

After adjusting for individual and contextual**-**level factors (model 3) education status of women, the total numbers of living children, ANC follow-up, region, and proportion of women who had ANC follow-up were found to have a statistically significant association with iron-folic acid intake among pregnant women in Ethiopia. Those primary educated pregnant women were 1.8 times more likely to take iron-folic acid as compared to those not educated [AOR **=** 1.83, 95% CI: (1.24, 2.74)]. Similarly, the odds of taking iron-folic acid were 2.75 times more in secondary educated pregnant women as compared to those not educated **[**AOR **=** 2.75, 95% CI: (1.57, 4.824)].

The odds of taking iron-folic acid among pregnant women who had greater than **5** living children were two times higher than those who had less than or equal to two children [AOR = 2.02, 95% CI: (1.25, 3.27)].

Those pregnant women who had ANC visits were 21 times more likely to take iron-folic acid as compared to women who hadn’t [AOR = 21.26, 95% CI: (13.56, 33.32)]. Similarly, those pregnant women who lived in a cluster with a high proportion of women who had ANC visits were 1.72 times more likely to take iron-folic acid as compared to women who lived in a cluster with a low proportion of women who had ANC visits [AOR = 1.72, 95% CI: (1.17, 2.54)]. But, the odds of taking iron-folic acid among pregnant women who lived i**n** Somali region were 56% less likely as compared to Dire Dawa [AOR = 0.44 0.73, 95% CI: (0.22, 0.87)] (Table 2).

**Table 2:** Individual and contextual-level factors associated with iron-folic acid intake in Ethiopia, mini-EDHS 2019 dataset

Variables	Category	COR (95% CI)	Null model	Model 2 AOR (95% CI)	Model 3 AOR (95% CI)
Age of mother in years	15–19	1		1	1
	20–24	1.71(3.10,8.46)		1.41 (0.73, 2.27)	1.34 (0.69, 2.59)
	25–29	1.73(1.03,2.91)		1.30 (0.66, 2.55)	1.25 (0.64, 2.48)
	30–34	1.40(0.84,2.33)		1.18 (0.59, 2.40)	1.08 (0.54, 2.21)
	35–39	0.94(0.56,1.58)		0.80 (0.37, 1.72)	0.73 (0.34, 1.59)
	40–44	1.07(0.55,2.08)		1.28 (0.48, 3.35)	1.12 (0.43, 2.93)
	45–49	0.36(0.14,0.90)		0.37 (0.11, 1.25)	0.30 (0.09, 1.03)
Religion	Orthodox	1		1	1
	Muslim	0.54(0.34,0.84)		0.64 (0.42, 0.96)	1.08 (0.61, 1.923)
	Protestant	0.65(0.40,1.02)		0.48 (0.30, 0.75)	0.72 (0.42, 1.27)
	Other	0.64(0.4,1.73)		1.18 (0.50, 2.77)	1.76 (0.72, 4.35)
ANC visit	No	1		1	1
	Yes	27.14(17.25,42.71)		23.96 (15.32, 3746)	**21.26 (13.56, 33.32)**
Wealth	Poorest	1		1	1
	Poorer	1.90(1.33,2.72)		1.16 (0.75, 1.77)	1.05 (0.68, 1.63)
	Middle	2.88(1.99,4.15)		1.66 (1.07, 2.57)	1.45 (0.93, 2.26)
	Richer	2.84(1.88,4.29)		1.33 (0.82, 2.16)	1.10 (0.67, 1.82)
	Richest	6.72(3.89,11.59)		1.70 (0.94, 3.07)	1.32 (0.5&, 3.12)
Marital status	Single/not living together	1		1	1
	Married/living together	1.36(0.73,2.50)		0.95 (0.50, 1.81)	0.95(0.5000–08121).
Number of living children	≤ 2	1		1	1
	3–5	0.78(0.59,1.01)		1.26 (0.85, 1.86)	1.29 (0.88, 1.92)
	> 5	0.63(0.47,0.85)		1.876 (1.15, 3.02)	**2.02 (1.25, 3.27)**
Education status of a mother	No education	1		1	1
	Primary	2.17 (1.62, 2.90)		1.87 (1.26, 2.78)	**1.83 (1.24, 2.74)**
	Secondary	5.12(3.10,8.46		2.64 (1.51, 4.61)	**2.75 (1.57, 4.82)**
	Higher	3.48(1.69,7.19)		1.69 (0.76, 0.38)	1.78 (0.81, 3.92)
Place of residence	Rural	1			1
	Urban	0.40(0.26,0.62)			0.01 (0.40, 1.65)
Region	Dire Dewa	1			1
	Tigray	2.44(1.35,4.420			2.00 (0.91, 4.39)
	Afar	0.40(0.20,0.78)			1.05 (0.53, 2.0)
	Amhara	1.27(0.69,2.31)			1.98 (0.89, 4.40)
	Oromia	0.54(0.29,1.00)			0.91 (0.48, 1.75)
	Somali	0.64(0.30,0.14)			**0.44 (0.22, 0.87)**
	Benishangul	0.61(0.30,1.21)			0.74 (0.3, 1.46)
	SNNP	0.47(0.26,0.86)			1.02 (0.46, 2.27)
	Gambela	0.56(0.28,1.11)			0.53 (0.23, 1.22)
	Harari	0.85(0.46,1.57)			0.86 (0.47, 1.56)
	Addis Ababa	1.15(0.63,2.10)			0.49 (0.23, 1.03)
Cluster ANC follow up	Low	1			1
	High	7.06(5.2,9.6)			**1.72 (1.17, 2.54)**
Cluster below middle wealth	Low	1			1
	High	0.34(0.24,0.48)			0.81 (0.51, 1.28)
Cluster above secondary	Low	1			1
	High	2.84(1.97,4.09)			0.93 (0.58, 1.49)

**Footnote**: 1 = Reference, Cluster ANC follow up = Proportion of women who had ANC in a cluster, Cluster below middle wealth = Proportion of women blow the middle wealth quartile in a cluster and Cluster above secondary = Proportion of women educated secondary and above in a cluster, COR = Crude Odds Ratio and AOR = Adjusted Odds Ratio

### Random effect

The results of multilevel logistic regression for random effects showed that there was a significant variation in taking iron-folic acid across the clusters (Table 3). The Intra-cluster correlation coefficients showed that 32.04% of the variation in taking iron-folic acid was related to community-level factors. The full model also showed that there is a statistically significant variation in taking iron-folic acid across communities or clusters. About 64.52% of taking iron-folic acid in clusters was explained in the full model. Besides, the MOR confirmed that taking iron-folic acid was attributed to community-level factors. The MOR for taking iron-folic acid was 3.26 in the empty model which indicated that there was variation between communities (clustering) (3.26 times higher than the reference (MOR = 1)). The unexplained community variation in taking iron-folic acid decreased to MOR of 2.02 when all factors were added to the model. This showed that when all factors are considered, the effects of clustering are still statistically significant in the full models (Table 3).

**Table 3:** Measure of variation on individual and contextual-level factors associated with iron-folic acid intake in Ethiopia, mini-EDHS 2019 dataset

Measure of variation	Model 1 (Null model)	Model 2		Model 3
Variance	1.55	0.68		0.55
Explained variance (PCV %)	Reference	56.13		64.52
Median odds ratio (MOR)	3.26	2.18		2.02
The intra-cluster correlation coefficient (ICC) in %	32.04	17.14		14.33
**Model fitness**				
Likelihood ratio test		0.0001	0.0085	

## Discussion

After adjusting for other variables and the effect of clustering, education status of women, the total numbers of living children, ANC follow-up, region, and living in a high proportion of women who had ANC follow-up were found to have a statistically significant association with iron-folic acid supplement intake among pregnant women in Ethiopia.

The result of this study indicated that as educational status increased, the odds of taking iron-folic acid supplementation among pregnant women also increased. The finding of this study is contrary to a study conducted in Ethiopia [21]. However, it is in line with a study conducted in Mecha district, Amhara, [25], and North Gondar, Ethiopia [26]. It is also in agreement with studies conducted in Addis Ababa [20, 27] and Southern Ethiopia [28] and a study conducted in India [29]. This may be due to the fact that education increases pregnant women’s knowledge, especially about the fates of micronutrient deficiency.

The odds of taking iron-folic acid supplementation among pregnant women who had greater than 5 living children were higher than those who had less than or equal to two children. This finding is consistent with a study conducted in North Gondar, Ethiopia [26]. It is also supported by the finding of a study conducted in Kenya [30]. This may be due to the presence of multiple exposures to its advantage and its risk as they have been exposed for medical care and counseling.

Those pregnant women who had ANC visits were more likely to take an iron-folic acid supplement as compared to women who hadn’t. This finding is supported by studies conducted in Ethiopia by using EDHS 2011 and 2016 respectively [21, 31]. Similarly, this finding is consistent with a study conducted in Tigray, Ethiopia [32], a systematic review and meta-analysis conducted in Ethiopia [33], and a study conducted in 22 countries [34]. The possible reason for the association may be due to the fact that women who had ANC visits may have the opportunity to know the benefits of iron-folic acid intake. In addition, the health care provider may provide counseling as to what will happen if they didn’t take it like postpartum anemia and neural tube defects [35, 36].

Those pregnant women who lived in a cluster with a high proportion of women who had ANC visits were more likely to take an iron-folic acid supplement as compared to women who lived in a cluster with a low proportion of women who had ANC visits. The possible association may be due to observational learning or as the women were living in a cluster with a high proportion of ANC visits, most of her intimate friends may be knowledgeable regarding it and there may be peer pressure even if she may not have the interest to take it.

Whereas, the odds of taking iron-folic acid supplementation among pregnant women who lived in Somali region were less likely as compared to Dire Dawa. This may be due to the fact that Somali region was one of the developing regions and there may be short access to media as compared to Dire Dawa. Despite different strengths: considering the clustering effect, and using a large sample size for analysis, it is not without limitations. Since this study takes secondary data, only small numbers of individual and contextual-level variables were included in the analysis as potential determinant factors for iron-folic acid intake. Furthermore, the finding of the study may also be prone to recall bias since the data was collected from women who gave birth five years before the survey.

## Conclusions

After adjusting for other variables and the effect of clustering, both individual and contextual factors were significantly associated with iron-folic acid intake during pregnancy. From individual-level factors: education status of women, the total numbers of living children, and ANC follow-up are significant and from contextual factors: region and living in a high proportion of women who had ANC follow-up were found to have a statistically significant association with iron-folic acid intake among pregnant women in Ethiopia. Promoting women’s education and maternal health services like ANC and intervention targeting Somali region would be the recalled area of the government.

## Data Availability

The datasets used and/or analyzed during the current study are available from the corresponding author on reasonable request.
